# Recurrent Transient Paresthesias After Revascularization for Moyamoya Disease: Hemodynamic Diagnostic Challenges in a Patient With Post-radioiodine Hypothyroidism

**DOI:** 10.7759/cureus.114767

**Published:** 2026-08-18

**Authors:** Tara Sanjabi, Ava Sanjabi, Lilia Olsick, Kian Memari, Sergio A Rodriguez, Nicholas A Trum, Walter Lajara-Nanson

**Affiliations:** 1 Dermatology, Touro College of Osteopathic Medicine Montana, Great Falls, USA; 2 Plastic and Reconstructive Surgery, Drexel University College of Medicine, Philadelphia, USA; 3 Medicine, Touro College of Osteopathic Medicine Montana, Great Falls, USA; 4 Family Medicine, Palmetto General Hospital, Hialeah, USA; 5 Research, Nova Southeastern University Dr. Kiran C. Patel College of Osteopathic Medicine, Davie, USA; 6 Internal Medicine, Benefis Health Systems, Great Falls, USA; 7 Neurology, Benefis Health Systems, Great Falls, USA

**Keywords:** cerebrovascular, cerebrovascular accident (stroke), encephaloduroarteriosynangiosis (edas), hemorrhagic stroke, ischemic cerebrovascular disease, moyamoya disease (mmd), puff of smoke appearance

## Abstract

Moyamoya disease (MMD) is a chronic progressive cerebrovascular arteriopathy characterized by stenosis or occlusion of the terminal internal carotid arteries (ICAs) with compensatory collateral vessel formation. Although surgical revascularization reduces ischemic risk, recurrent neurologic symptoms may persist and can create a diagnostic challenge when structural imaging remains stable. We report the case of a 31-year-old Black woman with a prolonged course of MMD complicated by prior hemorrhagic and ischemic cerebrovascular events who continued to experience recurrent transient unilateral paresthesias after bilateral direct superficial temporal artery (STA)-middle cerebral artery (MCA) bypasses and subsequent indirect burr-hole revascularization.

During the current presentation, she developed worsening left facial numbness, which was consistent with her established paresthesia pattern, and a severe headache without new focal deficits beyond baseline. Computed tomography angiography demonstrated unchanged chronic right-sided steno-occlusive disease with extensive collateralization and preserved distal flow, although diffusion-weighted MRI and dedicated perfusion imaging were not obtained during this encounter; prior MRI during similar episodes had shown no new infarction. Her course was further complicated by a history of post-radioiodine hypothyroidism with previously documented periods of thyroid instability, as well as inconsistent adherence to secondary prevention therapy and specialty follow-up.

This case highlights a post-revascularization diagnostic problem: distinguishing recurrent hemodynamic, migrainous, and ischemic symptoms in collateral-dependent moyamoya circulation can be difficult when large vessel imaging is stable. This distinction could not be definitively resolved in the current case because diffusion-weighted MRI and perfusion imaging were not obtained during the acute episode. It emphasizes the importance of timeline-based diagnostic reasoning, cautious interpretation of adherence history, and individualized long-term surveillance after revascularization.

## Introduction

Moyamoya disease (MMD) is a chronic, progressive steno-occlusive arteriopathy involving the terminal internal carotid arteries (ICAs) and the proximal anterior and middle cerebral arteries (MCAs), accompanied by the development of fragile basal collaterals that produce the characteristic 'puff of smoke' appearance on angiography [1,2]. Patients remain at risk for ischemic stroke, intracranial hemorrhage, transient ischemic attacks, and recurrent transient neurologic symptoms throughout the disease course [1,2].

Recent diagnostic criteria distinguish MMD, an idiopathic vasculopathy, from moyamoya syndrome, which occurs in association with secondary conditions such as Down syndrome, autoimmune disease, cranial irradiation, and thyroid disease [2]. Although historically associated with pediatric East Asian populations, MMD is now recognized worldwide with a substantial adult burden in the United States. Women are affected more often than men, and racial and ethnic differences in presentation have been described, including a higher incidence among Black patients compared with non-Hispanic White individuals [3,4].

Surgical revascularization, whether direct, indirect, or combined, remains a cornerstone of management for symptomatic disease, but it does not eliminate future neurologic risk [5,6]. Less often emphasized is the diagnostic challenge posed by recurrent transient symptoms after revascularization when vascular imaging appears stable, and diffusion imaging remains negative or unchanged. In such cases, clinicians must distinguish among transient ischemic attacks, migraine-related symptoms, and hemodynamic episodes occurring in collateral-dependent territories.

Here, our patient had stable large-vessel imaging and prior diffusion-negative imaging, illustrating the diagnostic reasoning required when transient unilateral paresthesias recur post-surgical intervention. Rather than attributing recurrence to a single factor, this report highlights how multiple contributors, such as patient-reported inconsistent medication adherence and a history of post-radioiodine thyroid instability, may lower the threshold for transient neurologic dysfunction in a patient with chronically vulnerable cerebral perfusion. What distinguishes this case is the recurrence of diffusion-negative sensory spells despite two revascularization strategies (direct, then indirect) and radiographically stable disease. This pattern receives comparatively little attention relative to acute stroke or hemorrhage after moyamoya surgery. However, this case’s contribution is diagnostic rather than mechanistic, illustrating how difficult it is to adjudicate among these differentials without imaging obtained at the time of symptoms. The association between thyroid disease and MMD is discussed here as another biologically plausible contributor.

## Case presentation

A 31-year-old Black woman with MMD diagnosed at age 23 presented to a community hospital emergency department with concern for an acute cerebrovascular event. Her past medical history was notable for a right basal ganglia hemorrhage, a prior minor ischemic infarct, chronic migraine without aura, dyslipidemia, Graves disease treated with radioactive iodine followed by hypothyroidism, iron deficiency anemia, and anxiety. She denied caffeine, alcohol, tobacco, and illicit drug use. She had a history of self-reported intermittent medication use and inconsistent specialty follow-up, although objective adherence metrics were not available.

**Figure 1:**
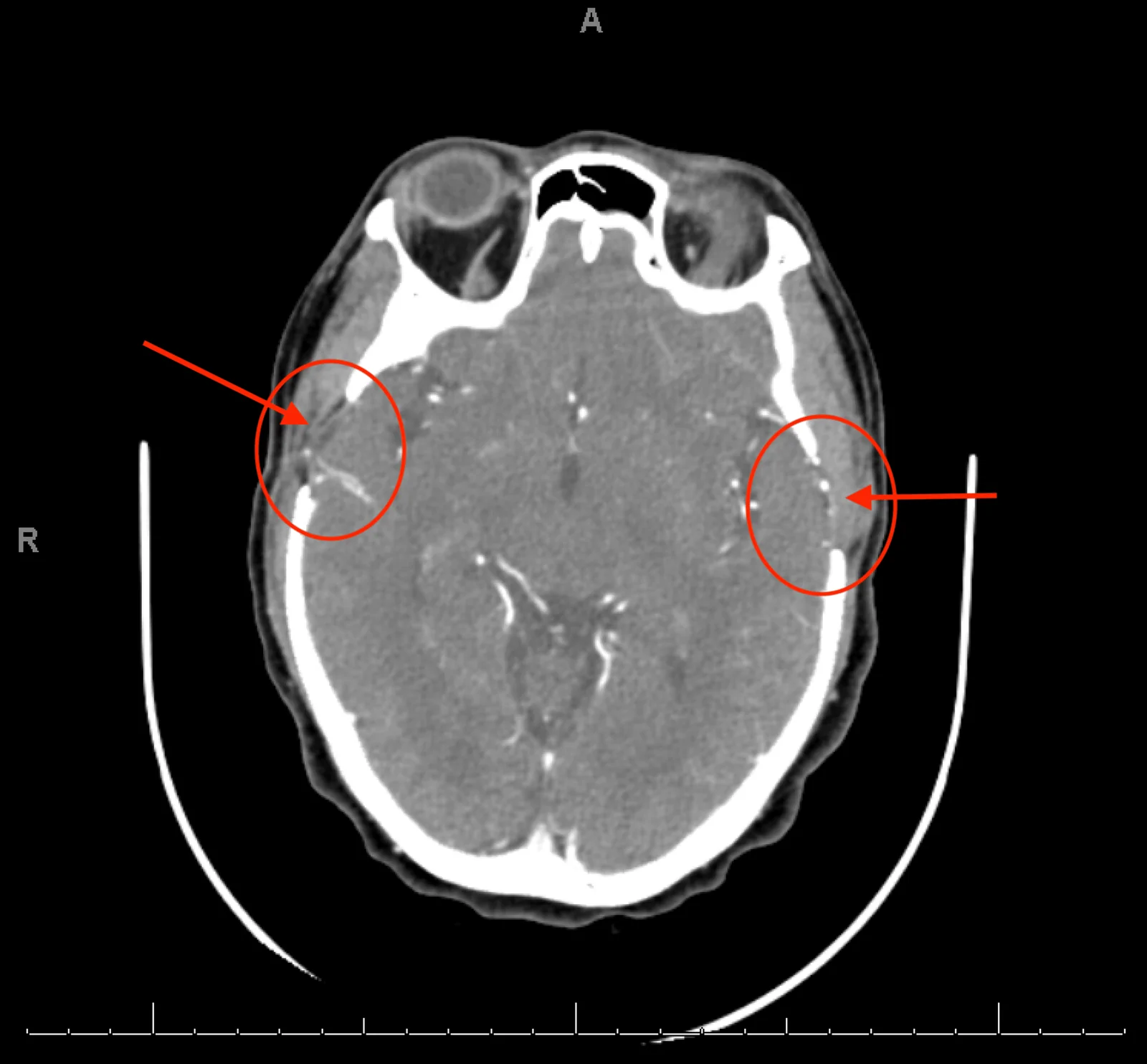
Noncontrast CT of the head demonstrating burr holes The circles in the image denote the locations of burr holes previously established in the patient during prior surgical interventions, performed as part of an indirect revascularization technique.

Her cerebrovascular course began at age 23 with direct surgical revascularization, consisting of staged bilateral superficial temporal artery (STA)-MCA bypasses, with the right side performed six months before the left side, as her initial revascularization procedure. At age 26, she had a right basal ganglia hemorrhage and presented with left cheek numbness that progressed to her jaw, then left arm and leg. In the months following, she continued to experience paresthesias, dizziness, and numbness. At age 27, because of the ongoing symptoms and concern for persistent perfusion vulnerability, she underwent additional bilateral indirect revascularization using burr-hole encephaloduroarteriosynangiosis (EDAS)-type procedures (Figure 1). Despite these interventions, from age 28 to 31, she continued to report intermittent stroke-like episodes characterized by visual blurring, light-headedness, left-sided hemiparesis, and a generalized sensation of heaviness, superimposed on chronic residual left-sided deficits from prior stroke. The recurrent paresthesia episodes form the basis of the 'recurrent transient paresthesias' referenced in this report's title.

At the current presentation at age 31, the patient reported an 8/10 frontal throbbing headache radiating intermittently to the vertex, worsened by sound, along with increased left facial numbness beginning the prior evening. This facial numbness was consistent in character and laterality with her prior recurrent paresthesia episodes, although it occurred alongside a headache more severe than her typical baseline migraine pattern, which is a relevant distinction to the differential discussed below. Two days earlier, she had been treated at urgent care for presumed sinusitis of six days' duration with over-the-counter decongestants. She stated that the current headache differed from her recent sinus symptoms and that the facial numbness had increased from baseline. Initial vital signs were blood pressure 140/92 mm Hg, temperature 98.5°F, heart rate 100 beats/minute, respiratory rate 16 breaths/minute, and oxygen saturation 98% on room air. Physical examination revealed no new focal neurologic deficits beyond her baseline chronic findings.

**Figure 2:**
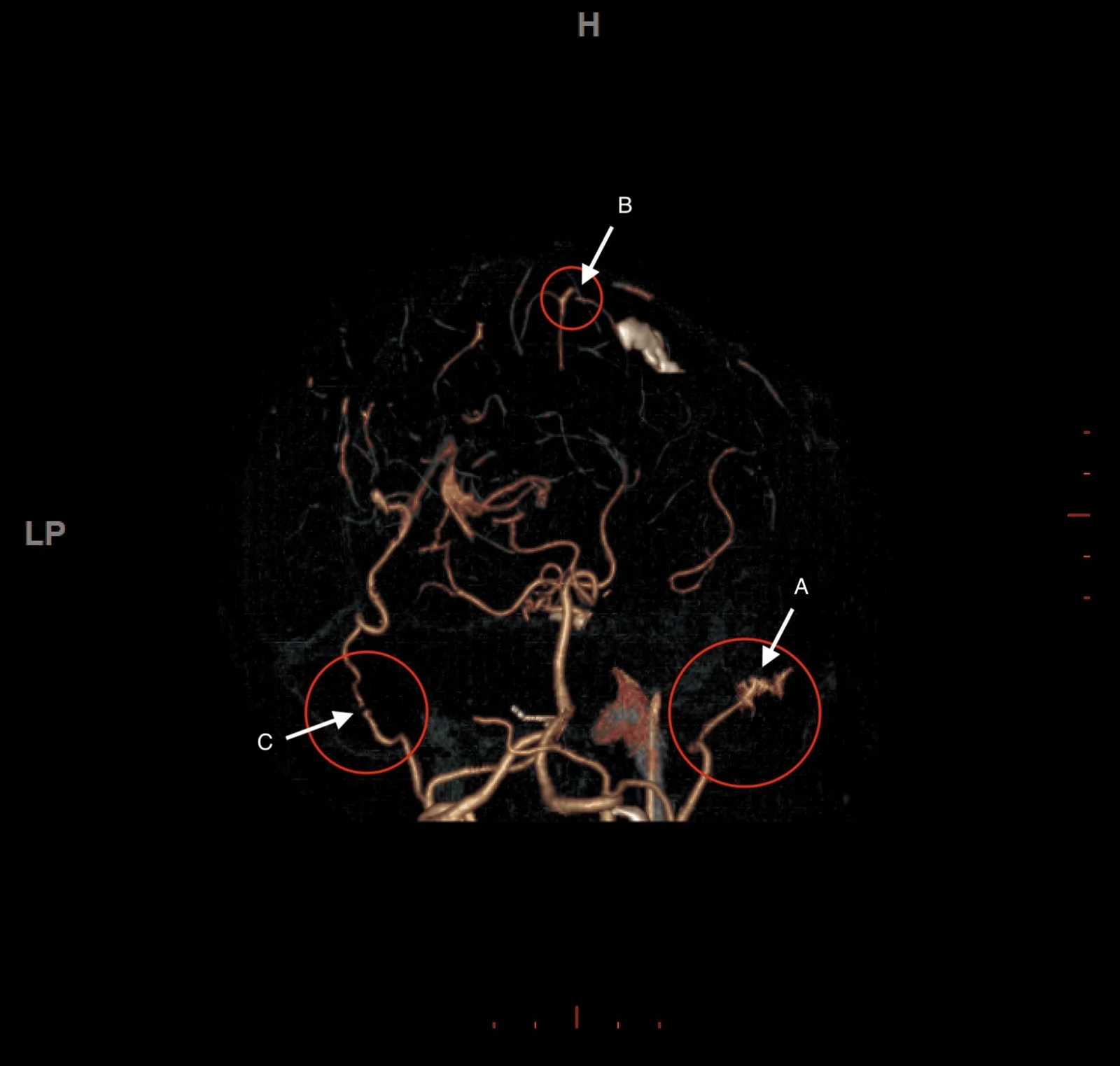
CTA of the head demonstrating characteristic moyamoya collaterals and progressive arterial stenosis A: The characteristic 'puff of smoke' appearance associated with MMD, indicative of the formation of fragile collateral vessels due to steno-occlusive changes in the ICAs. B and C: Varying degrees of stenosis observed within the arterial system, highlighting the pathological narrowing of vessels contributing to the progressive nature of MMD. CTA: Computed tomography angiography, MMD: Moyamoya disease, ICAs: Internal carotid arteries

Laboratory evaluation was notable only for mild hyponatremia (133 mmol/L) and mild hypokalemia (3.4 mmol/L). Thyroid function testing was not obtained during this encounter; her hypothyroidism was managed outpatient as an established chronic diagnosis and was stable on her most recent labs. Computed tomography angiography (CTA) of the head and neck demonstrated no interval change from prior imaging, with occlusion of the distal right ICA and proximal right MCA and an extensive collateral network compatible with MMD (Figure 2).

**Figure 3:**
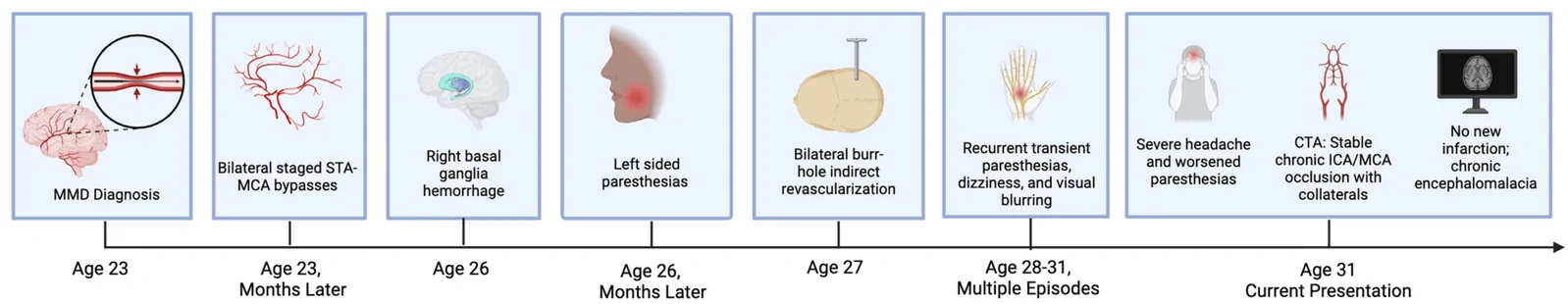
Clinical timeline of MMD progression and recurrent neurologic symptoms This is an overview of the patient's course from initial diagnosis through direct revascularization, basal ganglia hemorrhage, indirect revascularization, and current presentation of unilateral weakness and paresthesia with stable imaging. The patient's age at each event is shown in years; where two events occurred within the same age-year, their relative order and approximate interval are indicated by a qualifier to preserve anonymity. MMD: Moyamoya disease, CTA: Computed tomography angiography, STA: Superficial temporal artery, ICA: Internal carotid artery, MCA: Middle cerebral artery Illustration created by authors using BioRender.com (BioRender; Toronto, ON, CAN) without the use of generative AI tools.

Because of the current negative CTA and extensive prior imaging during similar episodes, diffusion-weighted MRI was not obtained during this admission. She received 1,000 mL of lactated Ringer's solution along with intravenous diphenhydramine and metoclopramide, improved symptomatically during observation, and was discharged with return precautions. The patient's clinical course is demonstrated in Figure 3.

During similar episodes, prior CTA demonstrated stable right intracavernous ICA and proximal M1 occlusion with a web-like collateral network and reliance on left-sided collateralization; prior serial MRI during multiple spells showed no new infarction but demonstrated encephalomalacia. Flow was preserved in the distal right M1 segment and in both the anterior and posterior cerebral arteries, with prominent collateralization along the course of the patent left MCA, consistent with MMD. These findings suggest chronic collateral-dependent cerebral perfusion rather than a new large-vessel occlusion.

After a minor infarct due to moyamoya vasculopathy, neurology started dual antiplatelet therapy (aspirin 81 mg and clopidogrel 75 mg daily) for 21 days, then continued clopidogrel 75 mg daily for secondary stroke prevention. This remained her regimen at presentation. The patient later reported taking antiplatelet therapy irregularly. However, because objective adherence data were unavailable, this was interpreted as a potential contributory factor rather than the primary explanation for recurrence.

## Discussion

Moyamoya disease remains a chronic and progressive steno-occlusive vasculopathy even after revascularization. This case presents an under-discussed post-revascularization dilemma in which recurrent transient neurologic symptoms may occur despite stable structural vascular imaging, prior negative diffusion studies during similar episodes, and multiple potential physiologic modifiers of cerebral perfusion. The principal diagnostic challenge in this case was distinguishing among transient ischemic attack, migraine-associated sensory symptoms, and hemodynamic transient neurologic episodes in a patient with chronically compromised cerebrovascular reserve after prior revascularization.

Transient ischemic attack was considered because of the focal unilateral paresthesias and prior ischemic history. However, a recurrent embolic or thrombotic event was less likely because there were no new deficits beyond baseline, no interval CTA changes, and diffusion-negative MRI during prior similar spells. Although a large infarct was ruled out, a small acute infarct cannot be excluded because diffusion-weighted MRI was not obtained during the current encounter.

Migraine-related symptoms were also plausible given her chronic migraine history, severe throbbing headache, phonophobia, and symptomatic improvement with diphenhydramine and metoclopramide. Her improvement after diphenhydramine and metoclopramide, both standard migraine treatments, supports a migraine or migraine-hemodynamic overlap mechanism for her headache. Despite a history of migraines, they are not likely the sole cause because of the recurrent stereotyped laterality of the sensory symptoms in the setting of known collateral-dependent circulation.

Hemodynamic transient symptoms were strongly considered because the patient had chronic right-sided steno-occlusive disease with extensive collateral reliance, recurrent diffusion-negative episodes, and possible physiologic stressors, including headache, recent illness, decongestant exposure, variable hydration status, mild electrolyte abnormalities, and a history of thyroid instability. Perfusion imaging, cerebrovascular reserve testing, transcranial Doppler evaluation, and catheter angiography were not obtained during this encounter, as presentation was considered clinically similar to prior episodes. The overall pattern favored transient hemodynamic vulnerability over new territorial infarction, though this remains a clinical hypothesis.

This patient's vascular substrate remained high risk despite a technically successful STA-MCA bypass and supplemental indirect EDAS/burr hole procedures to improve watershed perfusion. Formal assessment of graft patency by catheter angiography was not performed at this encounter; STA-MCA grafts were presumed patent based on preserved distal MCA flow on CTA. The CTA continued to show chronic right intracavernous ICA and proximal MCA occlusion with a web-like collateral network and dependence on collateral flow, while preserved distal perfusion suggested that symptoms could plausibly arise from marginal flow states rather than new vessel closure. This is consistent with the broader understanding that revascularization lowers but does not abolish ischemic or hemorrhagic risk in adult MMD [5,6]. The distinction between transient hemodynamic insufficiency or new infarction matters clinically because it shifts management toward careful trigger reduction, hydration, blood pressure optimization, and close neurovascular follow-up rather than reflexive attribution to irreversible ischemic progression [5,7].

Although the patient reported inconsistent use of antiplatelet therapy, the extent of nonadherence cannot be quantified due to the absence of pharmacy refill data or direct adherence monitoring. Recent meta-analyses have demonstrated that antiplatelet therapy may reduce the risk of platelet-mediated microthrombus formation at bypass anastomoses, lower transient ischemic attack risk, and support postoperative graft patency; however, evidence for reduction in ischemic stroke in MMD remains mixed [5,8,9]. Ergo, antiplatelet adherence is considered one of several plausible contributors to symptom control, rather than a unifying explanation. In this patient, adherence may have influenced risk, but spells often occurred without new diffusion-positive lesions and in the setting of hemodynamic stressors, supporting a hemodynamic component that antiplatelets alone would not address.

In this patient, endocrine instability may have been clinically relevant, as associations between MMD and thyroid disease, particularly Graves disease, are increasingly recognized and may reflect shared immune mechanisms [10,11]. Prior studies have reported elevated levels of soluble CD163 and CXCL5 in MMD, supporting an autoimmune and M2 macrophage-mediated component that may underlie its association with thyroid disease [10]. Furthermore, patients with MMD and thyroid disease have been reported to share genetic susceptibility through specific human leukocyte antigen (HLA) subgroups; high-resolution HLA typing identified HLA-DRB1*04:10 as a risk allele for MMD and associated with a higher frequency of thyroid disease, strengthening the endocrine-immunogenetic link [11]. In this patient, post-radioablative iodine hypothyroidism with previously documented periods of instability may have lowered the threshold for symptoms in flow-dependent territories, but this case does not establish a direct endocrine-vascular mechanism, as acute thyroid function was not tested at this specific encounter. Rather, it supports the practical importance of maintaining stable thyroid status as part of comprehensive long-term management. Classification of this patient's cerebrovascular disease as MMD rather than moyamoya syndrome reflects the onset of steno-occlusive disease prior to the diagnosis of any secondary syndromes such as Graves disease. 

This case highlights diagnostic limitations that can inform clinical practice. From a systems perspective, outcomes after surgical revascularization rely on adherence and routine follow-up. Guideline-concordant care emphasizes (1) sustained antiplatelet therapy in ischemic phenotypes; (2) lipid and blood-pressure optimization; (3) avoidance of hypotension/dehydration; (4) prompt evaluation of new focal deficits; and (5) scheduled neurosurgical/neurovascular follow-up to monitor graft patency and collateral maturation [5]. Perfusion imaging and diffusion-weighted MRI were not performed during the index presentation, and serial detailed neurologic examinations across each episode were limited in the available record. These gaps reduce certainty regarding the precise mechanism of each spell. In this case, inconsistent use of antiplatelets and statins, along with gaps in specialty follow-up, likely led to recurrent paresthesia. The overall pattern of recurrent sensory symptoms, stable CTA findings, and prior diffusion-negative MRI makes transient hemodynamic episodes and migraine plausible alternative explanations to repeated infarction, though confirmation would require perfusion and diffusion imaging obtained during each symptomatic episode.

## Conclusions

This case highlights the diagnostic complexity of recurrent transient neurologic symptoms after revascularization when large-vessel CTA imaging is stable, and prior MRI during similar episodes shows no new infarction. This case serves to prompt clinicians to obtain same-encounter diffusion and perfusion imaging where feasible so that hemodynamic, migrainous, and ischemic mechanisms can be distinguished with confidence. This case also highlights under-recognition outside of East Asian populations; clinicians should consider early vascular imaging in younger patients with recurrent focal symptoms or unexplained strokes. The practical lesson is that post-revascularization symptoms in MMD should not automatically be interpreted as either surgical failure, medication inconsistency, or thyroid instability. Timeline-based assessment, attention to baseline deficits, comparison with prior imaging, and management of reversible systemic factors are essential for accurate management and long-term surveillance.
